# Transport of symbiont-encoded cellulases from the gill to the gut of shipworms via the enigmatic ducts of Deshayes: a 174-year mystery solved

**DOI:** 10.1098/rspb.2022.1478

**Published:** 2022-11-09

**Authors:** Marvin A. Altamia, Daniel L. Distel

**Affiliations:** Ocean Genome Legacy Center, Department of Marine and Environmental Science, Northeastern University, Nahant, MA, USA

**Keywords:** *Bankia setacea*, Teredinidae, symbiosis, xylophagy, xylotrophy, bivalve

## Abstract

Shipworms (Bivalvia, Teredinidae) are the principal consumers of wood in marine environments. Like most wood-eating organisms, they digest wood with the aid of cellulolytic enzymes supplied by symbiotic bacteria. However, in shipworms the symbiotic bacteria are not found in the digestive system. Instead, they are located intracellularly in the gland of Deshayes, a specialized tissue found within the gills. It has been independently demonstrated that symbiont-encoded cellulolytic enzymes are present in the digestive systems and gills of two shipworm species, *Bankia setacea* and *Lyrodus pedicellatus*, confirming that these enzymes are transported from the gills to the lumen of the gut. However, the mechanism of enzyme transport from gill to gut remains incompletely understood. Recently, a mechanism was proposed by which enzymes are transported within bacterial cells that are expelled from the gill and transported to the mouth by ciliary action of the branchial or food grooves. Here we use *in situ* immunohistochemical methods to provide evidence for a different mechanism in the shipworm *B. setacea*, in which cellulolytic enzymes are transported via the ducts of Deshayes, enigmatic structures first described 174 years ago, but whose function have remained unexplained.

## 1. Introduction

Shipworms, wood-boring bivalves of the family Teredinidae, are among the most important wood consumers in marine environments [1]. Although capable of filter feeding [2], these wormlike mollusks are the only marine animals known to sustain normal growth and reproduction with wood as their sole source of solid food [2,3]. As a result of their wood-boring and wood-eating habits, shipworms cause extensive damage to wooden vessels, piers, fishing equipment and other man-made wooden structures in the sea, but also play an important beneficial role in marine carbon cycles by converting this recalcitrant material into more bioavailable forms [4]. Thus, understanding the shipworm's unusual dietary habits has important economic and ecological implications.

The digestive strategy used by shipworms to consume wood is extremely unusual. Most xylotrophic animals rely on the enzymatic activity of gut microorganisms to aid in the digestion of wood [4,5]. For example, wood-eating termites and cockroaches [6] (and to a lesser extent wood-eating beetles [7], fish [8] and mammals [9]) harbour lignocellulolytic microbes within specific gut compartments. These microbes are typically in direct contact with ingested wood and may contribute to wood digestion by supplying secreted or membrane-bound enzymes. Shipworms differ in that they digest wood in the caecum and the diverticula of the digestive glands, gut compartments that are largely devoid of microbes [10]. Although the caecum lacks a conspicuous microbiome, the gills of shipworms harbour dense populations of endosymbiotic gammaproteobacteria capable of producing cellulolytic enzymes [11–17]. These symbionts are housed in specialized cells called bacteriocytes in a tissue within the gills referred to as the gland of Deshayes [18].

The gland of Deshayes was first identified in 1848 by the geologist and conchologist, Gerard-Paul Deshayes [19] and was first described in detail in three shipworm species: *Xylotrya gouldi* (now *Bankia gouldi*)*, Teredo navalis* and *Teredo dilata* (now *Psiloteredo megatara*) by Charles Sigerfoos in 1907 [20]. Although Sigerfoos was ‘not able to even guess' a function for ‘this remarkable structure’, discoveries over the next century have shed considerable light. In 1973 Popham and Dixon [11] used transmission electron microscopy to demonstrate the presence of densely packed Gram-negative bacteria within the cells of the gland of Deshayes. Ten years later, Waterbury *et al*. [12] showed that a cellulolytic bacterium, later named *Teredinibacter turnerae* [21], could be isolated from the gills of numerous shipworm species. This bacterium was subsequently shown to be one of several *Teredinibacter* species present in the gland of Deshayes of shipworms [14,15,18,22–24]. Taken together, these observations suggested that these bacteria produce cellulases that might be transported to the gut to aid in wood digestion [12,15].

**Figure 1.**
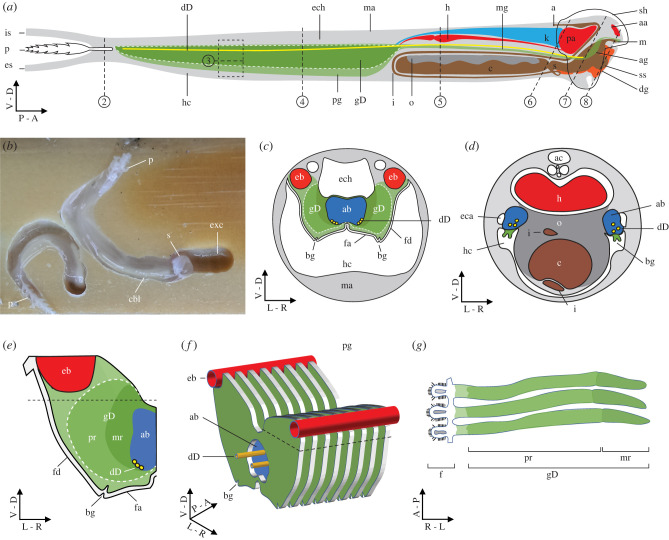
Anatomy of *Bankia setacea* (*a*) Diagram of the shipworm *B. setacea* viewed in sagittal section. Dashed lines 2–8 correspond to the locations and planes of section depicted in figures 2–8. (*b*) Two specimens of *Bankia setacea* in wood that has been dissected to expose the burrows. (*c,d*) Diagrams depicting transverse sections through the posterior gill (*c*) and middle gill (*d*) of *B. setacea* and corresponding to dashed lines 4 and 5 in (*a*), respectively. (*e*) An individual lamina (left side) removed from the posterior gill. (*f*) A three-quarter view of a portion of the posterior gill corresponding to the dashed box in (*a*) showing eight laminae in relation to the afferent and efferent branchial veins and ducts of Deshayes. (*g*) Three adjacent laminae in transverse section, corresponding to the black dashed lines in (*e*) and (*f*). White dashed lines in (*a*), (*c*) and (*e*) outline the location of the gland of Deshayse. aa, anterior adductor; ab, afferent branchial vein; ac, anal canal; ag, anterior gill; bg, branchial groove; c, caecum; cbl, calcified burrow lining; dD, duct of Deshayes; dg, digestive glands; eb, efferent branchial vein; es, exhalent siphon; exc, excavation chamber; eca, epibranchial canal; ech, epibranchial chamber; f, filament; fa, filament ascending limb; fd, filament descending limb; gD, gland of Deshayes; h, heart; hc, hypobranchial chamber; i, intestine; is, inhalant siphon; m, mouth; ma, mantle; mg, middle gill, mr, medial region of gland of Deshayes, o, ovary; p, pallets; pg, posterior gill; pr, peripheral region of the gland of Deshayes; s, stomach; sh, shell; ss, style sac. (Online version in colour.)

Indeed, symbiotic cellulase production and transport were later confirmed when it was shown that the most abundant cellulases detected in caecum of the shipworm *Bankia setacea* are encoded in the genomes of its gill endosymbionts [15]. In that study, it was suggested that the ducts of Deshayes might provide a plausible conduit for enzyme transport. These ducts were first described by Sigerfoos as minute vessels that travel anteriorly within the afferent branchial vein from the gland of Deshayes to a point near the anterior end of the esophagus or mouth (figure 1*a*). Similar ducts have been identified in *Teredo navalis* [25], *Nausitora hedleyi* [26], *Teredo furcifera* [26] and *Neoteredo reynei* [27].

More recently, the existence of the ducts of Deshayes and their relevance to enzyme transport in shipworms was questioned [16]. In that study, both symbiont- and host-encoded cellulolytic enzymes were detected in the gut of a second shipworm species, *Lyrodus pedicellatus* [16], however no evidence for the presence of the ducts of Deshayes was found. Instead, it was proposed that symbiont-encoded enzymes are transported from gill to gut within the cells of symbiotic bacteria that are shed from the gills and transported via ciliary action of the branchial groove to the mouth and digestive system where they augment the activity of host-encoded cellulases [16].

Here we use microscopy and *in situ* immunohistochemical methods to further explore possible pathways of symbiont-encoded enzyme transport in the shipworm *B. setacea*. To do so, we designed, produced and tested peptide antibodies against two symbiont-encoded lignocellulolytic enzymes identified as the major symbiont-encoded proteins in the caecum of *B. setacea* [15] and used these to search for anatomical features that might explain the presence of symbiont-encoded enzymes in the gut of this species*.* Based on the results of these experiments, we confirm the existence of the ducts of Deshayes in *B. setacea* and provide evidence for their role in transport of symbiont-encoded enzymes from gill to gut in this species.

## 2. Methods

### (a) Specimens

*Bankia setacea* specimens were collected under Oregon State Scientific Taking-Fish Permit no. 22153 in pine boards deployed at the Charleston Marina complex (43°20'48.5″N 124°19'22.3″W), Charleston, Oregon.

### (b) Antigen and antiserum preparation

Recombinant antigens and the corresponding antibodies were produced by a commercial laboratory (Genscript USA Inc., Piscataway NJ). Briefly, DNA sequences (electronic supplementary material, figure S1) of the predicted catalytic modules of two symbiont-encoded cellulases, GH5_53 (KJ861991) and GH6 (KJ861972), were synthesized with codon optimization, cloned in pET30a vector with a 6×-His tag added to the C-terminal, overexpressed in the recombinant host *E. coli* strain BL21 Star (DE3), subjected to Ni-NTA affinity purification, and injected to New Zealand rabbits. IgG antibodies that target the GH modules were affinity-purified from the immune sera and used for all the subsequent experiments.

### (c) Western blot analyses

To test the reactivity and specificity of the antibodies, SDS PAGE immunotransfer analyses (Western blots) were performed on protein extracts from shipworm tissues, cell-free culture supernatants from the symbionts *Teredinibacter waterburyi* str. Bs02^T^ and *T. purpureus* str. Bs12^T^, and the synthetic peptide antigens used to generate the antibodies. To prepare tissue protein extracts, gills, caecum and siphons were dissected, weighed and homogenized in 1 ml of ice-cold PBS buffer, pH 7.4 containing Protease Inhibitor Cocktail (Sigma P2714) and PMSF (Sigma P7626) at the manufacturer's suggested concentrations. Particulates were removed by centrifugation (13 000×*g*, 15 min, 4°C) followed by centrifugal filtration (Pall Nanosep MF GHP, 0.45 µm). To prepare protein extracts from culture supernatants, bacterial isolates were grown in liquid SBM medium with cellulose as the sole carbon source. Cells were then removed by centrifugation (10 000×*g*, 30 min, 4°C) and soluble proteins were concentrated using Vivaspin 20, 10 kDa MW cut-off centrifugal concentrators. Protein extracts from tissues and culture supernatants were then treated with non-reducing denaturing 4× treatment buffer (125 mM Tris · HCl buffer, pH 6.8, 50% glycerol, 2% SDS, 0.01% Bromophenol Blue), boiled for 3 min and separated on a 9% SDS-PAGE gel with or without 0.2% carboxymethylcellulose (CMC, Sigma C-5678). Approximately 26 µg and 14 µg of soluble protein was loaded per lane for culture supernatants and tissue extracts, respectively.

For Western blot analysis, proteins separated by SDS-PAGE were transferred to a PVDF membrane using a Bio-Rad Trans-Blot SD Semi-Dry Electrophoretic Transfer Cell following the manufacturer's recommended protocol. The membrane was blocked with 5% skimmed milk in TBS-T buffer (20 mM Tris · HCl buffer pH 7.6, 150 mM NaCl, 0.1% Tween 20) overnight at 4°C and subsequently incubated for 60 min at room temperature with either rabbit pre-immune sera (1 : 200) or affinity purified rabbit polyclonal IgGs generated against the GH5 or GH6 catalytic modules (2 µg ml^−1^) in 5% skimmed milk in TBS-T buffer. After incubation with the primary antibody, the membrane was washed 3 × 15 min each with TBS-T buffer and incubated with HRP-conjugated goat anti-rabbit IgG (ThermoFisher Scientific 31460) diluted at 1 : 5000 with 5% skimmed milk in TBS-T buffer, followed by 3 × 15 min washes with TBS-T buffer. Immunoreactive bands were visualized by incubating the membrane with DAB substrate (Millipore 281751) dissolved in TBS buffer with 0.3% H_2_O_2_.

### (d) Tissue preservation, embedding and sectioning

For microscopy, whole specimens of *B. setacea* were fixed in Bouin's fixative (Poly Scientific) overnight at 4°C, dehydrated through an ethanol series (30%, 50%, 70%, 15 min per step), then washed with 70% ethanol to eliminate residual picric acid. Fixed specimens were then embedded in paraffin, cut into 5 µm sections and mounted on glass slides at the Beth Israel Deaconess Medical Center Histology Core (Boston, MA). Adjacent sections were collected for histochemical staining (haematoxylin and eosin), immunohistochemical staining, and fluorescence *in situ* hybridization.

### (e) Immunohistochemistry

To detect GH5 and GH6 catalytic modules in tissues, slide-mounted sections were deparaffinized as described in [28] then incubated in 10 mM sodium citrate buffer, pH 6.0 with 0.05% Tween 20 for 4 h at 82°C and then 2 h at room temperature to promote protein refolding. Slides were rinsed briefly with TBS-T buffer and blocked with 5% skimmed milk in TBS-T buffer overnight at 4°C then incubated with 4 µg ml^−1^ primary antibody or 1 : 200 pre-immune serum in 1% BSA in TBS-T buffer for 2 h at room temperature. To assess non-specific binding of the secondary antibody, control slides were prepared without primary antibody and with pre-immune serum respectively. Slides were then washed 3× with TBS-T for 3 min each, followed by incubation with 5 µg ml^−1^ Alexa Fluor 633-conjugated goat anti-rabbit IgG (ThermoFisher Scientific A-21071) for 1 h in the dark at room temperature, then washed 3× with TBS-T buffer for 3 min each and air-dried. Slides were mounted using ProLong Diamond Antifade Mountant with DAPI (ThermoFisher P36966) and allowed to cure for 48 h. Sections were imaged on Zeiss LSM 880 confocal microscope (Beth Israel Deaconess Medical Center or Institute for Chemical Imaging of Living Systems, Northeastern University).

### (f) Fluorescence *in situ* hybridization

To detect bacterial symbionts in tissues, Cy5-labelled EUB338 Probe (5′-AGC CAT GCA GCA CCT GTC TC-3′) was applied to deparaffinized tissue sections in the presence of 30% formamide as described in [29]. An antisense version, EUB338 NON (5′-GAG ACA GGT GCT GCA TGG CT-3′) was applied as a negative control.

## 3. Results and discussion

### (a) Antibody specificity

In this investigation, we generated polyclonal antibodies targeting the cellulolytic catalytic modules of two glycoside hydrolases (GH) belonging to GH families 5 and 6 [30] and encoded in the genomes of the bacterial symbionts *Teredinibacter waterburyi* str. Bs02^T^ and *T. purpureus* str*.* Bs12^T^, respectively [15]. The purified anti-GH5 and anti-GH6 antibodies selectively bound the purified antigens on Western immunoblots (electronic supplementary material, figure S2). No labelling of the purified antigens was observed in pre-immune serum and no-protein controls. The anti-GH5 and anti-GH6 antibodies also bound to protein bands in extracts of the caecum contents and gill tissues of *B. setacea* (electronic supplementary material, figure S3). On Western immunoblots, the major antibody-reactive protein bands observed in gill and caecum extracts migrated just above and comigrated with the 46 kDa molecular weight standard, respectively. This is consistent with the previous observation that the full-length enzymes are proteolytically cleaved in the caecum of *B. setacea* to remove two carbohydrate-binding modules (CBM families 2 and 10) and their associated linkers, yielding fragments of 52.1 and 46.3 kDa, respectively [15]. No labelling of tissue extracts was observed in the pre-immune serum and no-protein controls and only trace labelling was observed in the symbiont-free siphon tissue extract. This trace labelling is likely due to residual fecal matter in the exhalent siphon.

The anti-GH5 and anti-GH6 antibodies also bound to discrete protein bands in cell-free supernatants of spent media in which *T. waterburyi* str. Bs02^T^ and *T. purpureus* str*.* Bs12^T^ were grown (electronic supplementary material, figure S4). This is consistent with the fact that genes encoding both proteins contain predicted bacterial secretion signals. We note however that both the anti-GH5 and anti-GH6 antibody-reactive protein bands in spent media migrate on SDS-PAGE gels with apparent molecular weights ≥100 kDa, larger than those predicted by their respective full-length secreted proteins (73.8 and 77.5 kDa, respectively) and the anti-GH5 antibody showed multiple bands. This likely reflects protein aggregation, incomplete denaturation or post-translational modification, e.g. glycosylation of the linkers which is commonly observed in bacterial cellulases and is known to decrease mobility in SDS PAGE gels [31–33]. Despite the discrepancy in the apparent molecular weight of the full-length proteins in spent media, no significant cross-reactivity was observed with proteins originating in the host genome or tissues. These results indicate that the antibodies described here may be useful for identifying and localizing symbiont-encoded GH5 and GH6 catalytic-module-containing proteins within the tissues of *B. setacea*.

### (b) Anatomy

The anatomy of *Bankia setacea,* highlighting the structure and organization of the gills, gland of Deshayes and ducts of Deshayes observed in this study, is diagrammed in figure 1. A more detailed description of the anatomy of *B. setacea* can be found in the online supplement.

### (c) Immunohistochemical enzyme localization

**Figure 2.**
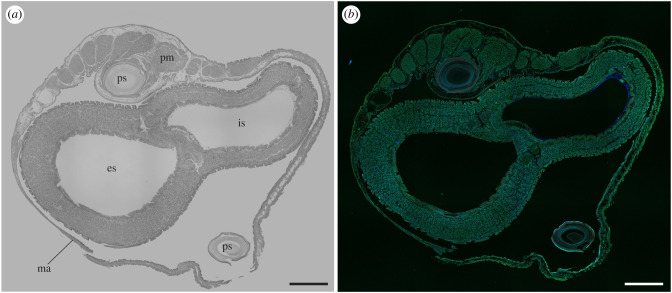
Immunohistochemical localization of anti-GH6 antibody in sections through the posterior end of a whole specimen of *B. setacea*. (*a*) A hematoxylin and eosin-stained tissue section and (*b*) a section stained immunohistochemically with an anti-GH6 antibody and Alexa Fluor 633-conjugated goat anti-rabbit IgG labelled secondary antibody (red). es, exhalent siphon; is, inhalant siphon; ma, mantle; pm, pallet musculature; ps, pallet stalk. Scale bars = 500 µm. Note that no antibody labelling is detectable in this section. (Online version in colour.)

**Figure 3.**
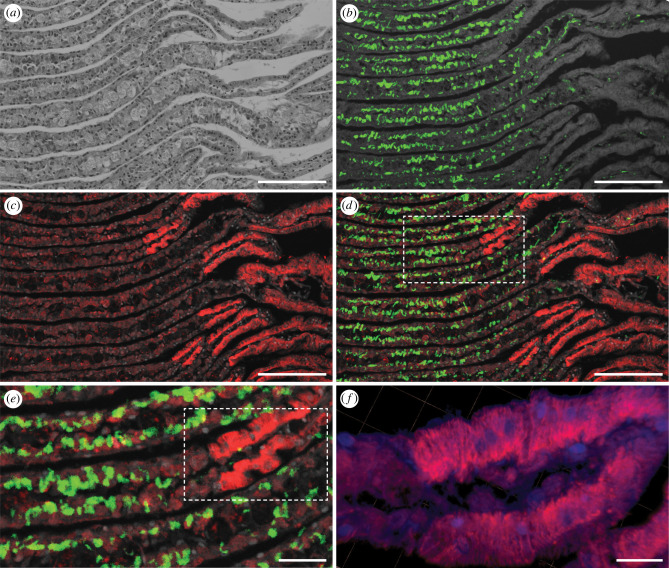
Immunohistochemical localization of anti-GH6 antibody binding in the laminae of the posterior gill of *B. setacea.* Frontal sections stained with (*a*) hematoxylin and eosin, (*b*) EUB338 bacteria-specific 16S rRNA-directed oligonucleotide probe (green), and (*c*) anti-GH6 antibody plus Alexa Fluor 633-conjugated goat anti-rabbit IgG labelled secondary antibody (red). (*d*) Merger of (*c*) and EUB338 signal from (*b*), (*e*) detail from boxed region in (*d*), (*f*) three-dimensional render of Z-stacked images of a tissue region similar to that seen in dashed box in (*e*), demonstrating the striated appearance of antibody staining within cells of the medial region of the gland of Deshayes. Nuclei in (*f*) are stained with DAPI (blue). Scale bars: (*a*)–(*d*) = 100 µm; (*e*) = 25 µm; (*f*) = 10 µm. (Online version in colour.)

**Figure 4.**
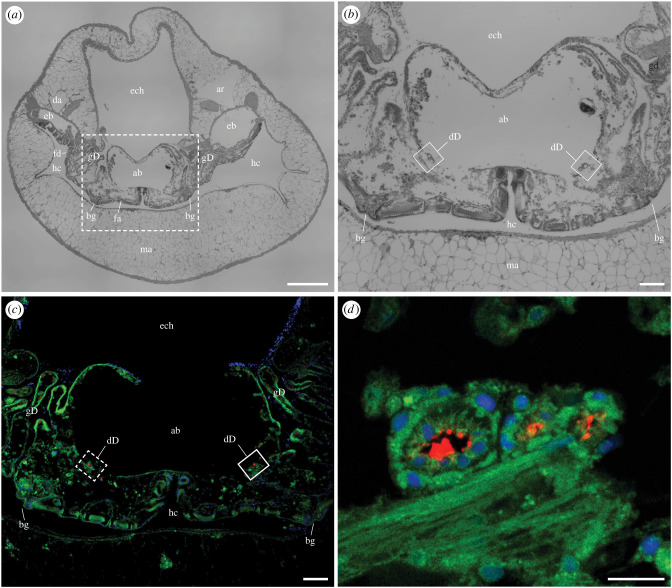
Immunohistochemical localization of anti-GH6 antibody binding in the lumen of the ducts of Deshayes within the posterior gill of *Bankia setacea.* (*a*) Transverse section through the posterior gill of *Bankia setacea* stained with hematoxylin and eosin, (*b*) Detail from the boxed region in (*a*) showing the location of the ducts of Deshayes (boxed), (*c*) Transverse section through the posterior gill of *Bankia setacea* labelled with anti-GH6 antibody plus Alexa Fluor 633-conjugated goat anti-rabbit IgG labelled secondary antibody (red) and DAPI (nuclei; blue), the green colour is due to tissue autofluorescence, (*d*) detail of dashed box in (*c*). ab, afferent branchial vein; ar, afferent renal vein; bg, branchial groove; da, dorsal artery; dD, duct of Deshayes; eb, efferent branchial vein; ech, epibranchial chamber; fa, filament ascending limb; fd, filament descending limb; gD, gland of Deshayes; hc, hypobranchial chamber, ma, mantle. Scale bars: (*a*) and (*b*) = 500 µm; (*c*) and (*d*) = 100 µm. (Online version in colour.)

**Figure 5.**
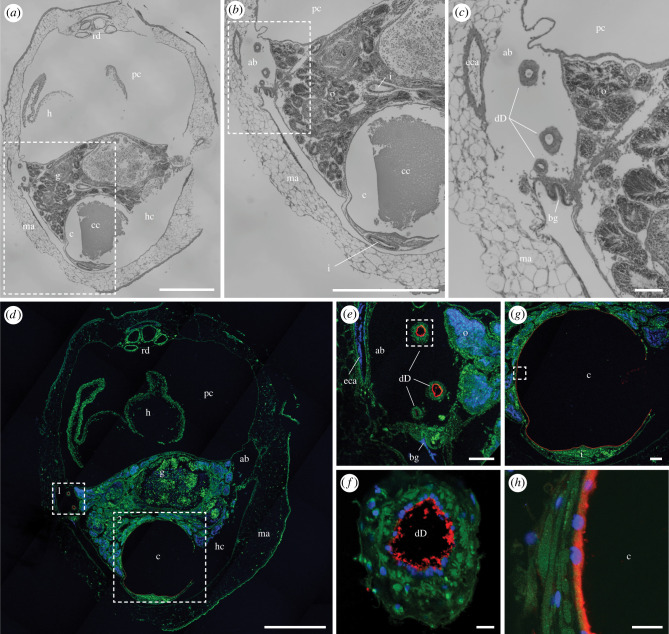
Immunohistochemical localization of anti-GH6 antibody binding in the lumen of the ducts of Deshayes and caecum in the posterior end of the visceral mass of *Bankia setacea*. Transverse section through the visceral mass and pericardial cavity of *Bankia setacea* stained with (*a–c*) hematoxylin and eosin and (*d–h*) anti-GH6 antibody plus Alexa Fluor 633-conjugated goat anti-rabbit IgG labelled secondary antibody (red) and DAPI (nuclei; blue), the green colour is due to tissue autofluorescence, (*b*) detail of dashed box in (*a*), (*c*) detail of dashed box in (*b*), (*e*) detail of dashed box 1 in (*d*), (*f*) detail of dashed box in (*e*), (*g*) detail of dashed box 2 in (*d*), (*h*) detail of dashed box in (*g*). ab, afferent branchial vein; bg, branchial groove; c, caecum; cc, caecum content; dD, ducts of Deshayes; eca, epibranchial canal; o, ovary; h, heart; hc, hypobranchial chamber; i, intestine; ma, mantle; pc, pericardial cavity; rd, renal ducts. Scale bars: (*a*) and (*d*) = 1000 µm; B = 500 µm; (*c*), (*e*) and (*g*) = 100 µm; (*f*) and (*h*) = 10 µm. (Online version in colour.)

**Figure 6.**
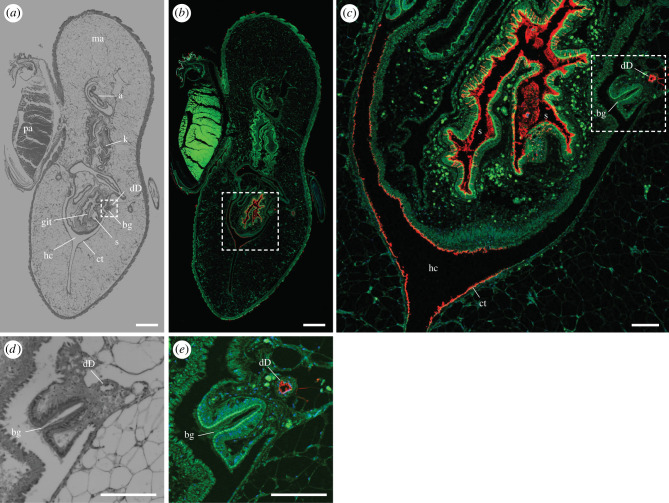
Immunohistochemical localization of anti-GH6 antibody binding in the posterior stomach and lumen of the ducts of Deshayes near the posterior shell margin of *Bankia setacea*. Transverse section through the kidney, anal canal, posterior adductor muscle and posterior end of the stomach near the cecal orifice stained with (*a,d*) haematoxylin and eosin and (*b,c,e*) anti-GH6 antibody plus Alexa Fluor 633-conjugated goat anti-rabbit IgG labelled secondary antibody (red), green colour is due to tissue autofluorescence, (*c*) detail of dashed box in (*b*), (*d*) detail of dashed box in (*a*), (*e*) detail of dashed box in (*c*). a, anus; bg, branchial groove; ct, ciliated tract; dD, duct of Deshayes; git, gastrointestinal typhlosole; hc, hypobranchial chamber; k, kidney; ma, mantle; s, stomach. Scale bars: (*a*) and (*b*) = 500 µm; (*c*), (*d*), and (*e*) = 100 µm. (Online version in colour.)

**Figure 7.**
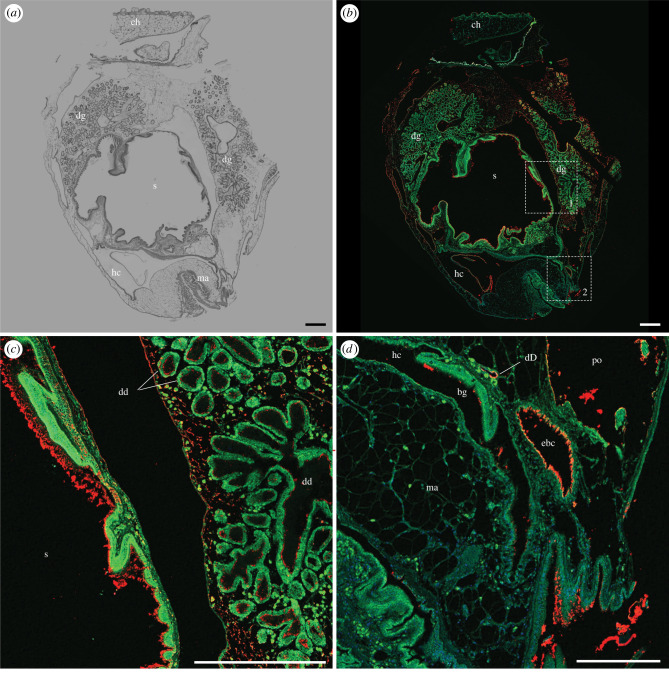
Immunohistochemical localization of anti-GH6 antibody in the anterior gut of *Bankia setacea*. Transverse section through the stomach, digestive gland, epibranchial canal, and hypobranchial chamber stained with (*a*) haematoxylin and eosin and (*b–d*) anti-GH6 antibody plus Alexa Fluor 633-conjugated goat anti-rabbit IgG labelled secondary antibody (red), green colour is due to tissue autofluorescence, (*c*) detail of dashed box 1 in (*b*). (*d*) detail of dashed box 2 in (*b*). Note that antibody labelling is evident on wood particles and surfaces within the gut and duct of Deshayes, but also on nearly all surfaces exposed to the excavation chamber. bg, branchial groove; ch, cephalic hood; dd, digestive diverticula; dD, ducts of Deshayes; dg, digestive gland; ebc, epibranchial canal; hc, hypobranchial chamber; ma, mantle; po, pedal opening; s, stomach. Scale bars: (*a*), (*b*), and (*c*) = 500 µm; (*d*) = 250 µm. (Online version in colour.)

**Figure 8.**
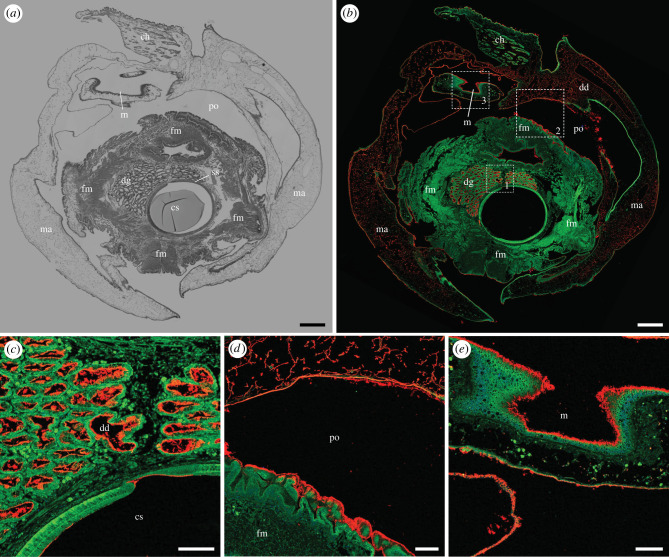
Immunohistochemical localization of anti-GH6 antibody binding near the anterior face of *Bankia setacea*. Transverse section through the stomach, mouth, digestive gland, style sac and foot, stained with (*a*) haematoxylin and eosin and (*b–e*) anti-GH6 antibody plus Alexa Fluor 633-conjugated goat anti-rabbit IgG labelled secondary antibody (red), green colour is due to tissue autofluorescence, (*c*) detail of dashed box 1 in (*b*). (*d*) Detail of dashed box 2 in (*b*) (*e*) Detail of dashed box 3 in (*b*). Note that antibody labelling is observed on wood particles and/or surfaces within the digestive diverticula, style sac and on nearly all surfaces exposed to the excavation chamber, including the mantle, mouth and foot. ch, cephalic hood; cs, crystalline style; dd, digestive diverticula; dg, digestive gland; fm, foot musculature; ma, mantle; m, mouth: po, pedal opening; ss, style sac. Scale bars: (*a*) and (*b*) = 500 µm; (*c*), (*d*) and (*e*) = 100 µm. (Online version in colour.)

To systematically explore the distribution of symbiont-encoded cellulases in *B. setacea*, we used immunohistochemical staining and confocal microscopy to examine the binding of anti-GH5 and anti-GH6 antibodies to tissue sections cut at intervals along the length of adult specimens from posterior to anterior. These sections show the siphons at the base of the pallets (figure 2), the posterior gill (figures 3 and 4), the posterior end of the middle gill, ovary, caecum and intestines (figure 5), the anterior end of the middle gill and posterior end of the stomach (figure 6), the anterior end of the stomach (figure 7), and the digestive glands, crystalline style, mouth and foot (figure 8). The orientations and locations of the sections shown in figures 2–8 are indicated by the correspondingly numbered dashed lines in figure 1*a*.

In this investigation, the observed patterns of tissue labelling were nearly indistinguishable for the anti-GH5 and anti-GH6 antibodies. Therefore, only sections labelled with the anti-GH6 antibody are displayed in figures 2–8. Adjacent sections showing anti-GH5 antibody labelling and anti-GH6 antibody labelling, as well as pre-immune serum, and no-secondary-antibody controls, are presented in the electronic supplementary material, figures S5–S11.

When either the anti-GH5 or anti-GH6 antibodies were applied to transverse sections cut near the posterior end of *B. setacea* (figure 2; electronic supplementary material, figure S5) no detectable labelling of tissue was observed. This region includes the mantle, siphons, pallets and pallet musculature. It contains no visceral organs, is free of bacterial symbionts, and does not participate in wood digestion.

By contrast, both the anti-GH5 and anti-GH6 antibodies labelled specific structures within the posterior gill, which lies within the mantle cavity just anterior to the siphons. Note that the gills of *B. setacea* are divided into three distinct regions, the large posterior gill which houses the gland of Deshayes, the threadlike middle gill composed largely of the branchial groove, and the tiny anterior gill. A detailed description of gill anatomy can be found in the online supplement. Figure 3 and electronic supplementary material, figure S6 show sagittal sections through the posterior gill in which the cells of the gland of Deshayes can be seen within the interlamellar tissue of multiple gill lamina. Labelling of the sections with a 16S rRNA-directed bacteria-specific fluorescent oligonucleotide probe (EUB338) showed that the gland of Deshayes is divided into two distinct regions (diagrammed in figure 1*g*). The first contains irregularly shaped bacteriocytes which appear green in figure 3*b*,*d*,*e* due to labelling of the bacterial symbionts with the EUB338 probe. The bacteriocytes lie in the peripheral portion of the laminae, closest to the filament. The medial region of each lamina, which lies closest to the afferent branchial vein, is composed of more regularly shaped cells that are symbiont-free. Figure 3*c–f* and electronic supplementary material, figure S6 show that both the anti-GH5 and anti-GH6 antibodies (red) bind specifically to bacteriocytes in the peripheral region, approximately co-localizing with staining due to the bacteria-specific EUB338 probe (figure 3*d*,*e*). The approximate co-location of the symbiont cells and the symbiont-encoded cellulases is consistent with previous observations that symbionts actively express these proteins within the bacteriocytes [15–17].

Surprisingly, however, more intense antibody labelling was observed in symbiont-free cells of the medial region of the lamina than in the symbiont-containing bacteriocytes themselves. This is indicated by the intense red fluorescence seen on the right side of each lamina in figure 3*c–f*. A three-dimensional rendering of Z-stacked images of antibody labelling within the medial cells (figure 3*f*) reveals a complex pattern of striations spanning the width of these cells.

While the gland of Deshayes is most easily visualized in sagittal sections, the ducts of Deshayes are more easily seen in transverse sections, as these ducts are very narrow in diameter and extend along the anterior–posterior axis (figure 1*a*,*c–f*). Figure 4*a* and *b* show a haematoxylin-eosin-stained transverse section through the posterior gill of *B. setacea* in which the ducts of Deshayes can be seen in ventrolateral positions against the right and left walls of the afferent branchial vein, in the same location as previously described in *Bankia gouldi*, *Teredo navalis* and *Psiloteredo megatara* [20]. Immunohistochemical staining with both the anti-GH6 and anti-GH5 antibodies (figure 4*c* and *d* and electronic supplementary material, figure S7) show labelling of amorphous material within the lumen and adhering to the interior walls of the ducts of Deshayes. Up to three distinct ducts are observed on each side of the afferent branchial vein in *B. setacea*.

The ducts of Deshayes were traced anteriorly within the common afferent branchial vein (figure 5*a–c*) as this vein exits the posterior gill and divides into right and left branches that pass anteriorly within the right and left mantle lobes toward the anterior gill (diagrammed in figure 1*d*). As observed in the posterior gill, both the anti-GH5 and anti-GH6 antibodies bind specifically to material within the lumen of the duct of Deshayes in the middle gill region (figure 5*d–f*; electronic supplementary material, figure S8). In addition to the lumen of the ducts, sections in this region also show antibody labelling of material adhering to the inner surface of the lumen of the caecum (figure 5*g*,*h*; electronic supplementary material, figure S8), a primary location of wood digestion in shipworms [15]. Notably, antibody labelling was not evident on the ciliated surface of the branchial groove in this region (figure 5*e*) or on any other tissues or surfaces, including those of the ovaries, pericardial chamber, kidneys and mantle.

A similar pattern of antibody labelling is seen in transverse sections through the body just posterior to the posterior shell margin (figure 6; electronic supplementary material, figure S9). These sections transect the anal canal, kidney, posterior end of the stomach near the cecal opening, branchial grooves and the ducts of Deshayes near the anterior end of the middle gill. Here again, antibody labelling is seen within the lumen of the duct of Deshayes and within the lumen of the gut adhering to the gut lining and to wood particles within the gut. Notably, anti-GH5 and anti-GH6 antibody labelling can also be seen on other body surfaces in this and more anterior sections. For example, both antibodies label material on the ciliated tracts within the hypobranchial chamber (figure 6*c*; electronic supplementary material, figure S9) and faint anti-GH5 labelling can be seen on the surface of the branchial groove (electronic supplementary material, figure S9D) in these sections. However, no bacterial cells could be detected on these surfaces using the EUB 338 bacterial probe (electronic supplementary material, figure S9F).

Still greater labelling of body surfaces is apparent in more anterior tissue sections. Figure 7 and electronic supplementary material, figure S10 show transverse sections passing through the anterior end of the stomach and digestive glands. As before, both antibodies label material adhering to the stomach lining and to wood particles within the stomach, as well as to material within the ducts and diverticula of the digestive glands, and within the lumen of the ducts of Deshayes. However, here and in more anterior sections, antibody labelling can also be seen on all surfaces of the animal that communicate with the fluid-filled excavation chamber, including the inner surface of the hypobranchial chamber, the lumen of the epibranchial canal, the surfaces of the branchial grooves, the external surfaces of the mantle and the matrix surrounding the lobules of the digestive gland.

Finally, figure 8 and electronic supplementary material, figure S11 show sections grazing the anterior end of the specimen where the foot and surrounding mantle tissue face into the excavation chamber. These sections transect the mantle, foot, and mouth as well as the anterior edge of the style sac and anterior lobe of the digestive gland. Here, both the anti-GH5 and anti-GH6 antibodies label the surface of the mouth, the interior surface of the style sac, wood particles within the lumen of the digestive diverticula, all exterior surfaces of the foot and mantle, and wood particles adhering to those surfaces. Interestingly, we also saw labelling of material apparently within the most anterior tissue of the mantle (figure 8*b*,*d*). Here the tissue appeared irregular and unconsolidated, lacking the organized architecture apparent in other parts of the mantle. Because it is unclear to what extent this appearance reflects the natural state of the tissue, tissue damage, or artefacts produced when sections graze an uneven surface, further investigation will be required to determine whether this labelling is within solid tissue or on the surface of a porous or damaged tissue.

### (d) Summary and conclusions

Our results confirm the presence of the ducts of Deshayes within the afferent branchial veins of *B. setacea* and show that they match in location and appearance to those described by Sigerfoos over a century ago. Both the anti-GH5 and anti-GH6 antibodies labelled material within the lumen of these ducts throughout their length, from the posterior gill to the anterior end of the branchial grooves near the mouth. Within the gland of Deshayes, both antibodies appeared to label tissues intracellularly. However, in other regions of the body, antibody labelling appeared to be primarily extracellular. This extracellular labelling included material within the lumen of the ducts of Deshayes, as well as in the lumen of the esophagus, stomach, digestive diverticula, caecum and intestines. No antibody labelling was observed on other surfaces of the body posterior to the posterior shell margin, which corresponds to the parts of the body that reside within the lined portion of the burrow. By contrast, antibody labelling was observed on most surfaces of the body anterior to the posterior shell margin, including the surfaces of the foot and mantle, hypobranchial chamber and epibranchial canal. This corresponds to the parts of the body that reside within the unlined, fluid-filled excavation chamber where wood boring and feeding occurs (figure 1*b*).

These observations are consistent with the proposed production of cellulolytic enzymes by symbiotic bacteria within the bacteriocytes of the gland of Deshayes and their subsequent transport via the ducts of Deshayes. Due to the small size of the ducts of Dehayes and the complex anatomy near the anterior face of *B. setacea,* we were unable to pinpoint the precise termini of the ducts. Nonetheless, we were able to trace the ducts from the posterior gills to within tens of microns of the mouth and to show antibody labelling throughout the ducts, digestive tract, on all surfaces of the animal that contact the excavation chamber, and on wood particles on these surfaces. We also observed labelling on the surfaces of the hypobranchial chamber and the epibranchial canals at their most anterior ends (figure 6*c* and 7*b,c*) both of which communicate with the excavation chamber. These results suggest that the ducts of Deshayes release symbiont-encoded cellulases at or near the mouth where these enzymes may enter the excavation chamber and adhere to wood particles via their carbohydrate-binding modules (CBMs). Those wood particles are then transported over the ciliated surface of the foot to the mouth. Once ingested, these particles are transported either into the diverticula of the digestive glands where they are phagocytosed and digested intracellularly or to the caecum where they are digested extracellularly [34].

Our results do not provide support for the recently proposed mechanism by which symbiotic bacteria are expelled from the surfaces of the gills and transported via ciliary action of the branchial grooves to the mouth [16]. Indeed, we saw little evidence of antibody labelling on the external surfaces of the gill or the branchial grooves, except at the most anterior end of the body where nearly all surfaces showed antibody labelling. Nor did we see evidence of abundant bacterial cells on the external surfaces of the gills or branchial grooves using a bacteria-specific fluorescent oligonucleotide probe (figure 3*b,d,e* and electronic supplementary material, figure S9E). Additionally, in previously published proteomic [15] and transcriptomic [16,17] analyses, the symbiont-encoded proteins detected in the shipworm gut were primarily secreted cellulases. We would expect that other abundant bacterial proteins would be detected in the gut as well if intact bacterial cells rather than secreted cellulases were transported from gill to gut. Finally, because bacterial cells do not typically store large quantities of enzymes, the proposed ciliary mechanism would require expulsion and transport of large numbers of bacterial cells, which would probably have been detected in this investigation and in previous investigations of the symbiont-containing tissues of shipworms.

The most surprising aspect of our investigation is the observation of intense antibody labelling within the symbiont-free medial cells of the gland of Deshayes (figure 3*c–f*). The location of this tissue between the bacteriocytes and the duct of Deshayes suggests that these cells may function to concentrate symbiont-made cellulolytic enzymes and transport them from the bacteriocytes to the ducts of Deshayes.

While these results are strongly suggestive, we cannot rule out the possibility that these polyclonal antibodies may cross-react with non-target proteins of host origin, including host-encoded cellulases. However, in addition to the characterizations performed in this investigation, several observations suggest that this is not the case. Recent transcriptomic and proteomic studies examined the expression of carbohydrate active enzymes in the gills, caecum, crystalline style and digestive glands of *Lyrodus pedicellatus*, a shipworm closely related to *B. setacea* [16,17]*.* These studies showed that the host genome encodes a variety of carbohydrate active enzymes, including several with predicted or demonstrated activity against cellulose. Significantly, however, no GH family 6 transcripts were found in the *L. pedicellatus* transcriptome, and although host-encoded GH family 5 transcripts were found, these were very lowly expressed. Furthermore, all host-encoded lignocellulose-active enzymes identified in the digestive gland of *L. pedicellatus* had highly similar homologues in the genomes of other bivalves. None showed compelling similarity to known bacterial proteins. Similarly, we could identify no close eukaryotic homologues of the polypeptides used to generate the anti-GH5 and anti-GH6 antibodies in this study in the GenBank database or in the published gill metagenomes of *B. setacea*. Furthermore, in this investigation, both the anti-GH5 and anti-GH6 antibodies produce nearly identical labelling patterns in host tissue sections. These two antibodies target distinct enzyme families that differ in amino acid sequence and protein fold. Therefore, it seems unlikely that these two distinct antibodies would coincidentally cross-react with eukaryotic host proteins that display the same pattern of distribution in host tissue.

Finally, we note that the mechanism we propose here does not answer a key question regarding the shipworm symbioses, namely, how symbiont-made enzymes are transported from their intracellular location within the bacteriocytes to an extracellular location from which they can be transported to the gut. Although our results suggest a role for the symbiont-free medial cells of the gland of Deshayes in this process, we are unaware of any analogous or homologous system that provides a precedent or explanation for this proposed function of the medial cells. For these reasons, we do not regard the mechanism of symbiont-encoded enzyme transport in shipworms as fully resolved, but instead demonstrate a role for the enigmatic and long-unexplained ducts of Deshayes in this process, and suggest a potentially novel transport mechanism in the medial cells of the gland of Deshayes that urgently merits further investigation.

## Data Availability

All data used in the described research are included in the main text of the manuscript or in the electronic supplementary material [35].

## References

[1] Turner RD. 1966 A survey and illustrated catalogue of the Teredinidae (Mollusca: Bivalvia). Cambridge, MA: Harvard University, The Museum of Comparative Zoology.

[2] Mann R, Gallager SM. 1985 Physiological and biochemical energetics of larvae of *Teredo navalis* and *Bankia gouldi* (Bartsch). J. Exp. Mar. Biol. Ecol. **85**, 211-228. (10.1016/0022-0981(85)90159-5)

[3] Gallager SM, Turner RD, Berg CJ. 1981 Physiological aspects of wood consumption, growth, and reproduction in the shipworm *Lyrodus pedicellatus* Quatrefages. J. Exp. Mar. Biol. Ecol. **52**, 63-77. (10.1016/0022-0981(81)90171-4)

[4] Distel DL. 2003 The biology of marine wood boring bivalves and their bacterial endosymbionts. In Wood deterioration and preservation*, ACS Symposium Series 845* (eds B Goodell, DD Nicholas, TP Schultz), pp. 253-271. Washington, DC: American Chemical Society Press.

[5] Cragg SM et al. 2015 Lignocellulose degradation mechanisms across the Tree of Life. Curr. Opin Chem. Biol. **29**, 108-119. (10.1016/j.cbpa.2015.10.018) 26583519 PMC7571853

[6] Brune A. 2014 Symbiotic digestion of lignocellulose in termite guts. Nat. Rev. Microbiol. **12**, 168-180. (10.1038/nrmicro3182) 24487819

[7] Scully ED, Geib SM, Carlson JE, Tien M, McKenna D, Hoover K. 2014 Functional genomics and microbiome profiling of the Asian longhorned beetle (*Anoplophora glabripennis*) reveal insights into the digestive physiology and nutritional ecology of wood feeding beetles. BMC Genom. **15**, 1-21. (10.1186/1471-2164-15-1096) PMC4299006 25495900

[8] Watts JEM, McDonald RC, Schreier HJ. 2021 Wood degradation by *Panaque nigrolineatus*, a neotropical catfish: diversity and activity of gastrointestinal tract lignocellulolytic and nitrogen fixing communities. Wood Degrad. Ligninolytic Fungi **99**, 209-238. (10.1016/bs.abr.2021.05.005)

[9] Pratama R, Schneider D, Boer T, Daniel R. 2019 First insights into bacterial gastrointestinal tract communities of the Eurasian Beaver (*Castor fiber*). Front. Microbiol. **10**, 1646. (10.3389/fmicb.2019.01646) 31428060 PMC6690062

[10] Betcher MA, Fung JM, Han AW, O'Connor R, Seronay R, Concepcion GP, Distel DL, Haygood MG. 2012 Microbial distribution and abundance in the digestive system of five shipworm species (Bivalvia: Teredinidae). PLoS ONE **7**, e45309. (10.1371/journal.pone.0045309) 23028923 PMC3447940

[11] Popham JD, Dickson MR. 1973 Bacterial associations in the teredo Bankia australis (Lamellibranchia, Mollusca). Mar. Biol. **19**, 338-340. (10.1007/BF00348904)

[12] Waterbury JB, Calloway CB, Turner RD. 1983 A cellulolytic-nitrogen fixing bacterium cultured from the gland of Deshayes in shipworms (Bivalvia: Teredinidae). Science **221**, 1401-1403. (10.1126/science.221.4618.1401) 17759016

[13] Luyten YA, Thompson JR, Morrill W, Polz MF, Distel DL. 2006 Extensive variation in intracellular symbiont community composition among members of a single population of the wood-boring bivalve *Lyrodus pedicellatus* (Bivalvia: Teredinidae). Appl. Environ. Microbiol. **72**, 412-417. (10.1128/AEM.72.1.412-417.2006) 16391072 PMC1352252

[14] Distel DL, Beaudoin DJ, Morrill W. 2002 Coexistence of multiple proteobacterial endosymbionts in the gills of the wood-boring bivalve *Lyrodus pedicellatus* (Bivalvia: Teredinidae). Appl. Environ. Microbiol. **68**, 6292-6299. (10.1128/AEM.68.12.6292-6299.2002) 12450854 PMC134422

[15] O'Connor RM et al. 2014 Gill bacteria enable a novel digestive strategy in a wood-feeding mollusk. Proc. Natl Acad. Sci. USA **111**, E5096-E5104. 25385629 10.1073/pnas.1413110111 PMC4250168

[16] Pesante G et al. 2021 Characterisation of the enzyme transport path between shipworms and their bacterial symbionts. BMC Biol. **19**, 233. (10.1186/s12915-021-01162-6) 34724941 PMC8561940

[17] Sabbadin F et al. 2018 Uncovering the molecular mechanisms of lignocellulose digestion in shipworms. Biotechnol. Biofuels **11**, 1-4. (10.1186/s13068-018-1058-3) 29527236 PMC5840672

[18] Distel DL, DeLong EF, Waterbury JB. 1991 Phylogenetic characterization and in situ localization of the bacterial symbiont of shipworms (Teredinidae: Bivalvia) by using 16S rRNA sequence analysis and oligodeoxynucleotide probe hybridization. Appl. Environ. Microbiol. **57**, 2376-2382. (10.1128/aem.57.8.2376-2382.1991) 1722662 PMC183578

[19] Deshayes GP. 1848 Histoire naturelle des mollusques. Exploration Scientific l' Algerie. Zoologie **1**, 35-76.

[20] Sigerfoos CP. 1907 Natural history, organization, and late development of the Teredinidae or ship-worms. In Bulletin of the bureau of fisheries (ed. Department of Commerce and Labor BoF), pp. 191-231. Washington, DC: Government Printing Office.

[21] Distel DL, Morrill W, MacLaren-Toussaint N, Franks D, Waterbury J. 2002 *Teredinibacter turnerae* gen. nov., sp. nov., a dinitrogen-fixing, cellulolytic, endosymbiotic gamma-proteobacterium isolated from the gills of wood-boring molluscs (Bivalvia: Teredinidae). Int. J. Syst. Evol. Microbiol. **52**(Pt 6), 2261-2269. 12508896 10.1099/00207713-52-6-2261

[22] Altamia MA et al. 2020 Secondary metabolism in the gill microbiota of shipworms (Teredinidae) as revealed by comparison of metagenomes and nearly complete symbiont genomes. mSystems **5**, e00261-20. (10.1128/mSystems.00261-20) 32606027 PMC7329324

[23] Altamia MA, Shipway JR, Stein D, Betcher MA, Fung JM, Jospin G, Eisen J, Haygood MG, Distel DL. 2020 *Teredinibacter waterburyi* sp. nov., a marine, cellulolytic endosymbiotic bacterium isolated from the gills of the wood-boring mollusc *Bankia setacea* (Bivalvia: Teredinidae) and emended description of the genus Teredinibacter. Int. J. Syst. Evol. Microbiol. **70**, 2388. (10.1099/ijsem.0.004049) 32100688 PMC7395619

[24] Altamia MA, Shipway JR, Stein D, Betcher MA, Fung JM, Jospin G, Eisen J, Haygood MG, Distel DL. 2021 *Teredinibacter haidensis* sp. nov., *Teredinibacter purpureus* sp. nov. and *Teredinibacter franksiae* sp. nov., marine, cellulolytic endosymbiotic bacteria isolated from the gills of the wood-boring mollusc *Bankia setacea* (Bivalvia: Teredinidae) and emended description of the genus *Teredinibacter*. Int. J. Syst. Evol. Microbiol. **71**, 004627. (10.1099/ijsem.0.004627) 33439117 PMC8346767

[25] Lazier EL. 1924 Morphology of the digestive tract of *Teredo navalis*. Univ. Calif. Publ. Zool. **22**, 455-474.

[26] Saraswathy M, Nair NB. 1971 Observations on the structure of the shipworms, Nausitora hedleyi, Teredo furcifera and Teredora princesae (Bivalvia: Teredinidae). Earth Environ. Sci. Trans. R. Soc. Edinb. **68**, 507-566. (10.1017/S0080456800014861)

[27] De Moraes DT, Lopes SGBC. 2003 The functional morphology of *Neoteredo reynei* (Bartsch, 1920) (Bivalvia, Teredinidae). J. Mollus Stud. **69**, 311-318. (10.1093/mollus/69.4.311)

[28] IHC deparaffinization protocol: Abcam. See https://www.abcam.com/protocols/ihc-deparaffinization-protocol (accessed on 1 June 2018).

[29] Fuchs BM, Pernthaler J, Amann R. 2007 Single cell identification by fluorescence in situ hybridization. Methods General Mol. Microbiol. **5**, 886-896.

[30] Lombard V, Golaconda Ramulu H, Drula E, Coutinho PM, Henrissat B. 2013 The carbohydrate-active enzymes database (CAZy) in 2013. Nucleic Acids Res. **42**, D490-D495. (10.1093/nar/gkt1178) 24270786 PMC3965031

[31] Knowles J, Lehtovaara P, Teeri T. 1987 Cellulase families and their genes. Trends Biotechnol. **5**, 255-261. (10.1016/0167-7799(87)90102-8)

[32] Langsford ML, Gilkes NR, Singh B, Moser B, Miller Jr RC, Warren RA, Kilburn DG. 1987 Glycosylation of bacterial cellulases prevents proteolytic cleavage between functional domains. FEBS Lett. **225**, 163-167. (10.1016/0014-5793(87)81150-X) 3121390

[33] Chung D et al. 2019 Glycosylation is vital for industrial performance of hyperactive cellulases. ACS Sustain. Chem. Eng. **7**, 4792-4800. (10.1021/acssuschemeng.8b05049)

[34] Nair NB, Saraswathy M. 1971 The biology of wood-boring teredinid molluscs. Adv. Mar. Biol. **9**, 335-509. (10.1016/S0065-2881(08)60345-4)

[35] Altamia MA, Distel DL. 2022 Transport of symbiont-encoded cellulases from the gill to the gut of shipworms via the enigmatic ducts of Deshayes: a 174-year mystery solved. Figshare. (10.6084/m9.figshare.c.6261908)

